# Enhancing methyldopa solubility via green supercritical fluid techniques using ethanol co-solvent

**DOI:** 10.1038/s41598-025-15596-3

**Published:** 2025-08-13

**Authors:** Hadil Faris Alotaibi, Suleiman Ibrahim Mohammad, Asokan Vasudevan, Suranjana V. Mayani, Suhas Ballal, Munthar Kadhim Abosaoda, Abhayveer Singh, Subhashree Ray, Atreyi Pramanik

**Affiliations:** 1 Department of Pharmaceutical Sciences, College of Pharmacy, Princess Nourah Bint, AbdulRahman University, Riyadh, 11671 Saudi Arabia; 2 Electronic Marketing and Social Media, Economic and Administrative Sciences, Zarqa University, Zarqa, Jordan; 3 Research follower, INTI International University, 71800 Negeri Sembilan, Nilai, Malaysia; 4 Faculty of Business and Communications, INTI International University, Negeri Sembilan, 71800 Malaysia; 5 Department of Chemistry, Faculty of Science, Marwadi University Research Center, Marwadi University, Rajkot, Gujarat India; 6 Department of Chemistry and Biochemistry, School of Sciences, JAIN (Deemed to be University), Bangalore, Karnataka India; 7 College of pharmacy, the Islamic University, Najaf, Iraq; 8 College of pharmacy, the Islamic University of Al Diwaniyah, Al Diwaniyah, Iraq; 9 Centre for Research Impact & Outcome, Chitkara University Institute of Engineering and Technology, Chitkara University, Rajpura, 140401 Punjab India; 10 Department of Biochemistry, IMS and SUM Hospital, Siksha ’O’ Anusandhan (Deemed to be University), Bhubaneswar, 751003 Odisha India; 11 School of Applied and Life Sciences, Division of Research and Innovation, Uttaranchal University, Dehradun, Uttarakhand India

**Keywords:** Correlation, Binary, Ternary, Solubility, Carbon dioxide, Biochemistry, Biotechnology, Medical research, Nanoscience and technology

## Abstract

The aim of this study is to investigate the solubility of methyldopa in supercritical carbon dioxide. Solubility was examined at temperatures of 338, 328, 318, and 308 K, in both the presence and absence of a co-solvent. Specifically, ethanol was evaluated as a co-solvent. Semi-empirical and empirical approaches, as well as the Peng-Robinson equation, were applied to analyze the experimental data. The mole fraction (solubility) of methyldopa was found to range from 0.078 × 10^−4^ (0.21 g/l) to 1.082 × 10^−4^ (3.38 g/l) in the binary system and from 0.405 × 10^−4^ (1.38 g/l) to 3.143 × 10^−4^ (11.33 g/l) as well as from 1.225 × 10^−4^ (4.60 g/l) to 8.979 × 10^−4^ (34.94 g/l) at 1 and 3 mol % respectively. The findings indicated that incorporating a co-solvent, particularly ethanol, resulted in a substantial enhancement in solubility. The maximum enhancement was recorded in the methyldopa-ethanol-CO_2_ system at 12 MPa and 338 K, where the solubility was found to be 15.70 times higher than in pure supercritical carbon dioxide. The models employed for data evaluation exhibited robust correlations with the experimental outcomes. Among these models, the Soltani-Mazloumi model exhibited the strongest correlation with the experimental solubility data.

**Supplementary Information:**

The online version contains supplementary material available at 10.1038/s41598-025-15596-3.

## Introduction

Methyldopa is an antihypertensive medication primarily used to treat high blood pressure, particularly in pregnant women. It acts as a centrally acting alpha-2 adrenergic agonist, stimulating alpha-2 receptors in the brain and decreasing sympathetic nervous system activity. This lowers vascular resistance. Once in the brain, the drug converts into its active form, α-methylnorepinephrine, which inhibits the production of neurotransmitters, such as dopamine and norepinephrine. This disrupts baroreceptor signaling. Methyldopa is commonly used to treat preeclampsia and gestational hypertension. These effects have been shown to reduce blood pressure and promote placental development in the early stages of pregnancy. However, it is important to note that these effects may also increase the risk of depression, thought to occur through related biological pathways. Methyldopa’s gastrointestinal absorption is incomplete and variable, with bioavailability after oral administration ranging from 8 to 62%^1,2^. Bioavailability refers to the rate and extent to which a pharmaceutical compound is absorbed into the bloodstream. It plays a crucial role in determining the effectiveness of medications. Inadequate absorption may necessitate higher doses, which can pose economic and health risks. One key factor affecting bioavailability is a drug’s solubility in bodily fluids; smaller particle sizes improve dissolution rates and absorption^3–5^. Traditional methods of reducing particle size include sublimation, evaporation, milling, and cryogenic spraying. Recently, supercritical fluid techniques have gained attention for their ability to improve control over particle size and eliminate impurities. Supercritical carbon dioxide (scCO₂) is particularly effective in producing uniform, small particles while maintaining the stability of heat-sensitive compounds. This innovative method shows promise in improving drug solubility and bioavailability, which can lead to better therapeutic outcomes^6–9^. In recent decades, there has been an increased focus on applying supercritical fluids to drug processing. This approach has been shown to effectively reduce particle size, control particle morphology, and remove contaminants. Consequently, it has been shown to enhance the dissolution rates and bioactivity of pharmaceuticals. Primary techniques in this field include supercritical anti-solvent processes such as supercritical anti-solvent gas, solvent enhanced extraction, and solvent-enhanced dispersion. Additionally, significant advancements have been made in the rapid expansion of supercritical solutions. A substantial body of research has investigated the solubility of various APIs in scCO₂. This research has led to significant advancements in drug formulations, improving solubility and bioavailability^10–15^.

However, determining the solubility of substances in CO₂ is time-consuming and costly, requiring significant resources. To address this issue, several predictive models have been developed. These models encompass empirical and semi-empirical relationships, as well as equations of state in both cubic and non-cubic forms. Additionally, advanced methodologies such as machine learning algorithms and artificial neural networks have been used to improve these models’ predictive capabilities. The models aim to establish relationships between solubility under supercritical conditions and various parameters to enable more efficient predictions^16–26^.

Models based on thermodynamic equations often require estimations of more parameters, which can complicate their application. To overcome this, alternative models have been proposed by researchers such as Bian et al.^27^, Bartle et al.^28^, MST^29,30^, Del Valle and Aguilera^31^, Garlapati – Madras^32^, K-J^33^, and González et al.^34^. These models use observational data, theoretical frameworks, and relationships between solute solubility, density, pressure, and temperature. Integrating solubility data with carbon dioxide density yields a comprehensive system for predicting solubility in supercritical conditions.

Despite its advantages, scCO₂ faces challenges, particularly in solubilizing polar and hydrophilic solutes. This can hinder the efficient extraction and processing of certain active pharmaceutical ingredients (APIs). Often, this limitation requires the use of co-solvents, which alter the polarity of scCO₂ and significantly increase solute solubility. Common co-solvents include acetone, ethanol, dimethyl sulfoxide (DMSO), menthol, and methanol, which are typically used in small quantities with scCO₂^35–42^.

The Sodeifian-Sajadian^43^, Soltani-Mazloumi^44^, Garlapati-Madras^45^, and Jouyban et al.^46^ models are the most effective semi-empirical models for predicting the solubility of solids in scCO_2_ with cosolvents. They are particularly useful for predicting the solubility of pharmaceuticals, such as ketoconazole, rivaroxaban, and aripiprazole, in ternary systems. The Jouyban model is notable for its high accuracy and predictive power despite its minimal data requirements. The Sodeifian-Sajadian model is flexible, and the Soltani-Mazloumi model is user friendly and requires limited data. The Garlapati-Madras model showed promise for ternary systems but was less widely used. These models are essential for process optimization in industries such as pharmaceuticals. However, their empirical nature limits extrapolation, and challenges such as data dependency and parameter optimization persist.

To identify the most effective supercritical fluid process, it is crucial to understand how the solubility of the drug substance changes with temperature and pressure variations. This information is essential for designing and developing pharmaceutical applications. Currently, data on the solubility of methyldopa in CO₂ and ethanol is lacking. The present study aims to improve our understanding of methyldopa’s solubility behavior under scCO₂ conditions, considering the impact of ethanol as a cosolvent in both its absence and presence. This experiment explored dynamic solubility parameters within an expanded temperature range of 308 to 338 K and pressure range of 12 to 30 MPa. We then analyzed the solubility of methyldopa with respect to several operational parameters, including pressure, temperature, and the presence of ethanol at concentrations of 1 and 3 mol percent. To correlate the solubility data effectively, we employed Peng-Robinson equation (PR) and a variety of density models, including the Méndez-Santiago and Teja (MST, Sodeifian-Sajadian, Soltani-Mazloumi, Jouyban et al., Madras and González et al. models. Each model was meticulously evaluated for its predictive accuracy using statistical measures such as the correlation coefficient (R2), and the average absolute relative deviation (AARD). By comparing these metrics, we aim to establish a robust understanding of methyldopa solubility dynamics in both ternary and binary systems, ultimately enhancing the application of scCO2 in pharmaceutical processing.

## Materials and methods

### Materials

Methyldopa with a CAS Registry Number of 41372-08-1 and a purity level of 99.0%, was procured from Sigma-Aldrich in Germany. The carbon dioxide utilized in the present investigation was 99.99% (Table 1.).

**Table 1:** Properties of methyldopa.

Component	Methyldopa
Formula	C_10_H_13_NO_4_
IUPAC name	(S)−2-amino-3-(3,4-dihydroxyphenyl)−2-methyl-propanoic acid
M_w_ (g/mol)	211.21
Structure	

### Methods

#### Solubility measurement method

**Fig. 1:**
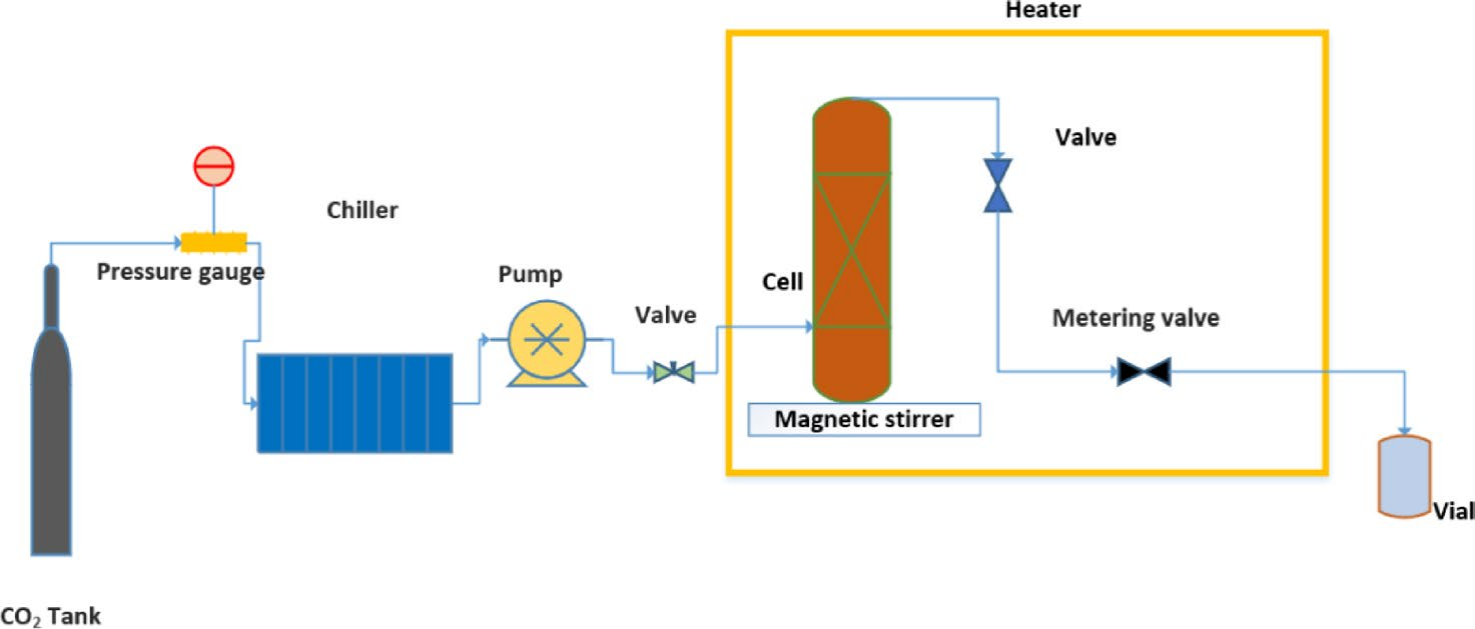
The schematic representation illustrates the apparatus utilized in the measurement of solubility.

The solubility of methyldopa was assessed using a method that combined a gravimetric technique (see Fig. 1). The experimental configuration was engineered to operate at pressures up to 40 MPa and temperatures up to 473 K, and the equilibrium vessel had a capacity of 100 mL. CO₂ was introduced into the solubility cell by a pump (Model 305, Gilson, France). that gradually increased the internal pressure in 0.1 MPa increments. This process culminated in a maximum pressure of 40 MPa. After the desired CO₂ pressure was attained, the flow into the cell stabilized. Then, ethanol was injected directly into the bottom of the cell at concentrations ranging from one to three%. A calibrated pressure gauge (WIKA, Germany), verified prior to the experiment, was used to monitor the pressure within the cell. Additionally, a control system that included a precise thermometer was implemented to maintain the system temperature within the specified range with an accuracy of ± 1 K. The drug was carefully molded into small, 6-millimeter tablets that was covered by glass wool and tissue. The tablets were then exposed to scCO₂ for four hours at a constant pressure and temperature while being continuously stirred at 400 rpm (see Figure S1). Preliminary tests were conducted on the equilibrium cell to determine if equilibrium had been achieved. These preliminary tests were conducted to determine the equilibrium state time (see Supplementary Table S1). After the four-hour exposure period, the cell was rapidly depressurized to ambient atmospheric conditions. The remaining amount of the drug was weighed using an analytical balance with a precision of 0.01 mg. Finally, the mole fraction of the drug was determined using a formula based on the initial and final masses of the drug.

1 $$\:{\text{m}}_{\text{e}}={\text{m}}_{\text{i}}-{\text{m}}_{\text{f}}\:$$

2 $$\:\text{M}\text{o}\text{l}\text{e}\:\text{o}\text{f}\:\text{d}\text{r}\text{u}\text{g}=\frac{{\text{m}}_{\text{e}}}{{\text{M}}_{\text{w},\text{d}\text{r}\text{u}\text{g}}}$$

3 $$\:{y}_{2}=\frac{\text{M}\text{o}\text{l}\text{e}\:\text{o}\text{f}\:\text{D}\text{r}\text{u}\text{g}\:}{(\text{M}\text{o}\text{l}\text{e}\:\text{o}\text{f}\:\text{d}\text{r}\text{u}\text{g}+\text{M}\text{o}\text{l}\text{e}\:{\text{o}\text{f}\:\text{C}\text{O}}_{2})}$$

4 $$\:S=\frac{\rho\:\times\:\:{M}_{solute}{\times\:\:y}_{2}}{{M}_{C{O}_{2}}\times\:\left(1-{y}_{2}\right)}.$$

#### Solubility models

- **Semiempirical equations**.

Mendez-Santiago and Teja^30^ developed a significant correlation combining the Clausius-Clapeyron equation and their proprietary formulations. This correlation incorporates sublimation pressure as a key variable. This advanced correlation includes four adjustable parameters that enable the analysis of interdependent factors, such as temperature, density, co-solvent composition, and solubility in ternary systems involving a solute, a solvent, and a co-solvent. Mendez-Santiago and Teja used this comprehensive approach to provide deeper insights into solubility behavior in supercritical fluid systems and enhance predictive accuracy for practical applications.

5 $$\:Tln\left(\frac{{y}_{2}^{{\prime\:}}P}{{P}_{ref}}\right)\:={a}_{0}+{a}_{1}{\rho\:}_{1}+{a}_{2}T+{a}_{3}{y}_{3}$$

Sodeifian-Sajadian^43^ focused on creating a model to correlate the solubility of APIs in carbon dioxide with the addition of a cosolvent. To ensure robustness, the model was trained by four adjustable parameters, representing the minimum requirements for effective calibration. This work builds on foundational research by scientists like Chrastil^47^and González et al.^34^, who explored the complexities of solute behavior in supercritical fluids.

$$\:\text{l}\text{n}\left({y}_{2}^{{\prime\:}}\right)\:={(a}_{0}+\frac{{a}_{1}{\rho\:}_{1}}{T})ln\left({\rho\:}_{1}\right)+{a}_{2}{\rho\:}_{1}+{a}_{3}\text{l}\text{n}\left({y}_{3}P\right)$$

González et al.^34^ introduced a thermodynamic model rooted in Chrastil’s research^47^, integrating a relationship between mole fraction and co-solvent concentration with the logarithmic dependence of solubility on solvent density. This method has proven highly effective in correlating solubility in non-entrained supercritical fluids, particularly in systems where the co-solvent significantly enhances solubility due to strong solute-co-solvent interactions. The model suggests that solute, co-solvent, and solvent can form clusters or solvate complexes, boosting solubility. However, it also highlights the impact of temperature, noting that higher temperatures can reduce solubility, potentially leading to inaccuracies in systems where the co-solvent acts merely as an additive without significant solubility-enhancing effects.

6 $$\:ln\left({y}_{2}^{{\prime\:}}\right)\:={a}_{0}ln\left({\rho\:}_{1}\right)+{a}_{1}ln\left({y}_{3}\right)+\frac{{a}_{2}}{T}+{a}_{3}$$

In a recent development, Soltani-Mazloumi^48^ introduced a five-parameter experimental model designed to estimate the solubility of solids in scCO₂ with a co-solvent. This model rigorously evaluates correlations among variables like temperature, density, pressure, and other factors influencing solubility in supercritical systems. Expanding on earlier work by Hozhabr et al.^49^, the model identifies three critical relationships: a linear relationship between the natural logarithm of solubility (ln y₂’) and the natural logarithm of the co-solvent mole fraction (ln y₃); a nonlinear relationship integrating ln y₂’, temperature, density, and a linear correlation between ln y₂’ and ln y₃, representing the cosolvent mole fraction.

7 $$\:ln\left({y}_{2}^{{\prime\:}}\right)={a}_{0}+\frac{{a}_{1}}{T}+\frac{{a}_{2}}{T}{\rho\:}_{1}-{a}_{3}{ln}\left(P\right)+{a}_{4}{ln}\left({y}_{3}{\rho\:}_{1}T\right)$$

Jouyban et al.^50^ proposed a sophisticated model requiring at least six experimental data points to accurately estimate the solubility of organic compounds in scCO₂ and co-solvent. Using advanced interpolation techniques across varying temperatures and pressures, this model stands out for its precision and user-friendly design, making it more accessible than other empirical equations and equations of state. Its combination of accuracy and ease of use makes it a valuable tool for researchers and practitioners in the field.

8 $$\:{ln}\left({y}_{2}^{{\prime\:}}\right)={a}_{0}+{a}_{1}{y}_{3}+{a}_{2}{\rho\:}_{1}+{a}_{3}{P}^{2}+{a}_{4}PT+\frac{{a}_{5}T}{P}+{a}_{6}ln{\rho\:}_{1}$$

Finally, Garlapati and Madras^32^ also made significant contributions with their 2010 equation, which extends the Jouyban et al.^50^ model. This equation incorporates seven variables linking the solubility of high-molecular-weight substances in scCO_2_ to critical factors such as temperature, scCO_2_ density, and co-solvent concentration. It is versatile, accommodating systems both with and without co-solvents, thereby broadening its applicability.

9 $$\:\text{ln}\left({y}_{2}^{{\prime\:}}\right)={a}_{0}+{a}_{1}ln\left({\rho\:}_{1}\right)+{a}_{2}{\rho\:}_{1}+\frac{{a}_{3}}{T}+{a}_{4}ln\left(T\right)\:+{a}_{5}ln\left({y}_{3}\right)+{a}_{6}ln\left({y}_{3}{\rho\:}_{1}T\right)$$

b) **Peng Robinson**.

In this study, the PR was employed to model the system. Equation (10), which describes the solubility of methyldopa in scCO_2_, has been adapted to incorporate the PR model for calculating fugacity coefficients.

The equation employed to estimate the mole fraction (y_2_) of methyldopa in scCO_2_ at a given temperature and pressure is as follows:

10 $$\:{y}_{2}=\frac{{P}_{2}^{sub}\left(T\right)}{P}\frac{{\varnothing\:}_{2}^{sat}\left(T\right)}{{\varnothing\:}_{2}(T,P,y)}exp\left[\frac{{v}_{2}^{s}(P-{P}_{2}^{sub}\left(T\right))}{RT}\right]$$

As demonstrated in Eq. 10, the fugacity coefficient ($\:{\varnothing\:}_{2}^{sat}\left(T\right)$) is a critical factor in the analysis. The sublimation pressure of methyldopa, denoted as ($\:{P}_{2}^{sub}$), was determined through the application of the Grain-Watson methodology, a well-established approach in the field. Concurrently, the Immirzi-Perini^51^ and the PR were employed to calculate the molar volume ($\:{v}_{2}^{s}=335.4\:\frac{{cm}^{3}}{mol}$) of methyldopa.

11 $$\:RTln\:{\varnothing\:}_{i}=-RTlnZ+{\int\:}_{V}^{\infty\:}\left[{\left(\frac{\partial\:P}{\partial\:{n}_{i}}\right)}_{T,V,{n}_{j}\ne\:{n}_{i}}-\frac{RT}{V}\right]dV$$

Wali et al.^52^ reported on the critical and physicochemical properties of methyldopa. The thermophysical properties of methyldopa were T_b_ = 844.5 K T_c_ = 1177.3 K, and P_c_ = 2.45 MPa. The acentric factor was estimated to be ω = 0.558 using the Ambros-Walton method. This section aims to provide a comprehensive description of the solubility data. Consequently, the PR model was selected for this study.

12 $$\:P=\frac{RT}{v-b}-\frac{a\left(T\right)}{v\left(v+b\right)+b\left(v-b\right)}$$

The attraction (*a*) and co-volume (*b*) constants were determined by applying the vdW2 as delineated in Eqs. (13) and (14), respectively.

13 $$\:{a}_{m}=\sum\:_{i}\sum\:_{j}{x}_{i}{x}_{j}{a}_{ij}$$

14 $$\:{b}_{m}=\sum\:_{i}\sum\:_{j}{x}_{i}{x}_{j}{b}_{ij}$$

The interaction parameters (*a*_*ij*_ and *b*_*ij*_) were calculated using the Eqs. (15) and (16).

15 $$\:{a}_{ij}={\left({a}_{i}{a}_{j}\right)}^{0.5}\left(1-{k}_{ij}\right)$$

16 $$\:{b}_{ij}=\left(\frac{{b}_{i}+{b}_{j}}{2}\right)\left(1-{l}_{ij}\right)$$

## Results and discussion

**Fig. 2:**
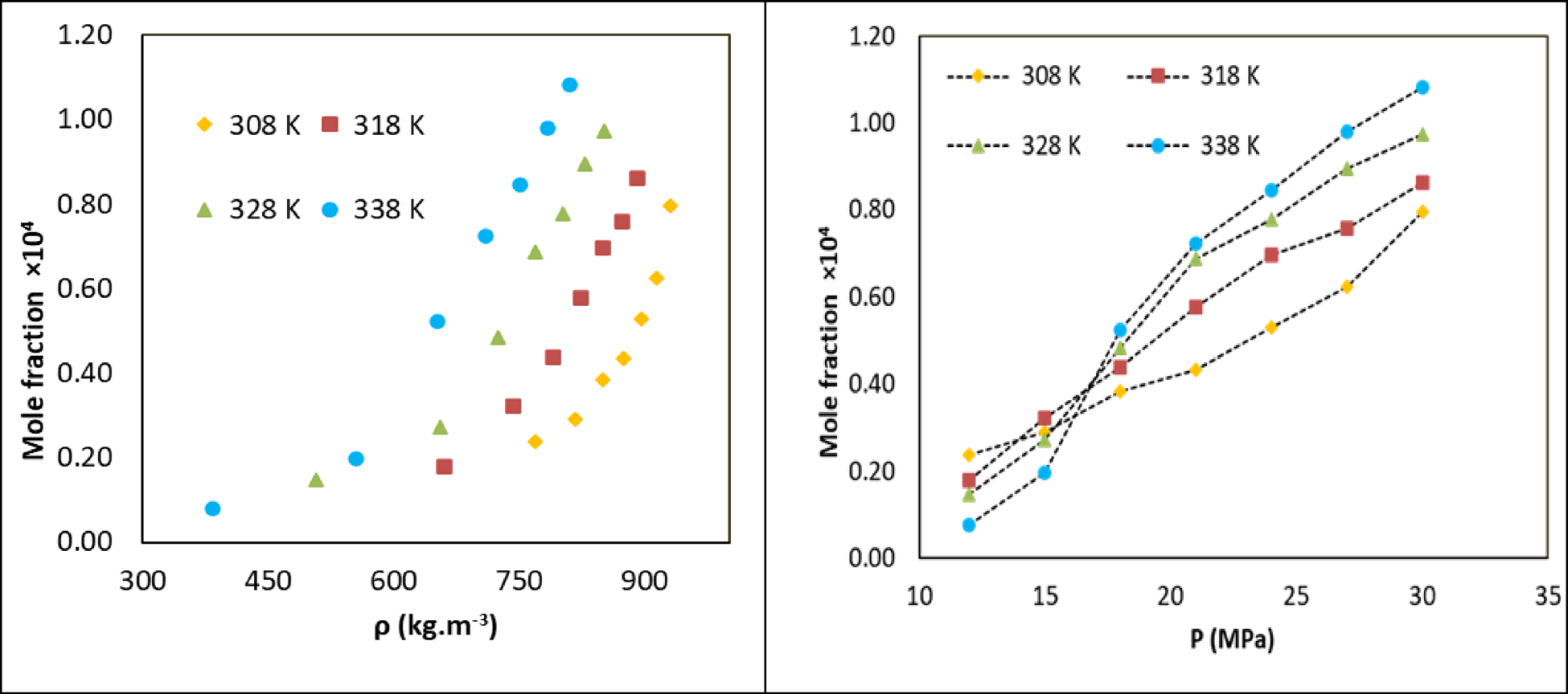
The methyldopa mole fraction in CO_2_ vs. pressure and density.

The present study constitutes an experimental investigation of the solubility of the pharmaceutical methyldopa in scCO₂ in ternary systems, with ethanol. The findings from these studies are presented in Table 2 and depicted in Fig. 2. The experiments were conducted at temperatures of 308, 318, 328 and 338 K and pressures of12, 15, 18, 21, 24, 27 and 30 MPa. The density of scCO₂ was obtained from the National Institute of Standards and Technology (NIST), ensuring an accurate depiction of the solvent’s characteristics. To ensure the reliability of the experimental results, each solubility measurement was conducted on three separate occasions, thereby ensuring a relative standard deviation of less than 4%.

**Table 2:** The experimental of Methyldopa solubility in scCO_2_.

Temperature (K)	Pressure (MPa)	Density of CO_2_ (kg/m^3^)	Binary (CO_2_ + methyldopa)		
			Mole fraction (y_2_) × 10^4^	Standard Deviation × (10^4^)	Solubility (g/l) *× 10*
308	12	768.4	0.238	0.009	0.88
	15	816.1	0.290	0.011	1.14
	18	848.9	0.385	0.015	1.57
	21	874.4	0.434	0.017	1.82
	24	895.5	0.529	0.021	2.28
	27	913.7	0.624	0.025	2.74
	30	929.7	0.795	0.032	3.55
318	12	659.7	0.179	0.007	0.57
	15	743.2	0.323	0.013	1.15
	18	790.2	0.439	0.017	1.66
	21	823.7	0.577	0.023	2.28
	24	850.1	0.697	0.028	2.84
	27	872.0	0.759	0.030	3.18
	30	890.9	0.863	0.034	3.69
328	12	506.9	0.148	0.006	0.36
	15	654.9	0.273	0.011	0.86
	18	724.1	0.484	0.019	1.68
	21	768.7	0.688	0.027	2.54
	24	801.9	0.778	0.031	3.00
	27	828.5	0.896	0.036	3.57
	30	850.8	0.975	0.039	3.98
338	12	384.2	0.078	0.003	0.14
	15	555.2	0.197	0.008	0.52
	18	651.2	0.523	0.021	1.63
	21	709.7	0.724	0.029	2.47
	24	751.2	0.845	0.034	3.05
	27	783.3	0.981	0.039	3.69
	30	809.6	1.082	0.043	4.21

Standard uncertainties are u(T) = 0.1 K; u(p) = 0.1 MPa.

In this study, the solubility of methyldopa was initially measured in CO_2_ (binary system). A comparison of the data with the findings reported in the 2024 paper by Wali et al.^52^ reveals that the average error is less than 4% (see Figure S2). The results indicate that methyldopa solubility generally increases with pressure, which enhances the solvating power of scCO₂.

The K-J model, which incorporates three adjustable parameters, proved highly effective in analyzing solubility data. This is evident from its AARD values of 10.10 (Chrastil), 11.41 (Bartle), 8.54 (K-J), and 10.51 (MST). Figure S3 shows strong correlation and consistency for supercritical CO_2_-solid solubility. To verify the reliability of these models, a series of self-consistency tests were conducted. These tests involved applying linear regression to the experimental data to establish solubility relationships. The observed linearity helped assess the models’ internal consistency. Among the models, the K-J approach exhibited the highest R² value (0.983), followed by MST (0.971), Bartle et al. (0.951), and Chrastil (0.981). This indicates the K-J approach’s superior extrapolation capability (see Supplementary Table S2 and S3). Solvation heat (ΔH_sol_) was calculated using the following equation: ΔH_sol_ = ΔH_tota_l – ΔH_vap_, where ΔH_total_ is obtained via the Chrastil model and ΔH_vap_ is derived from the Bartle et al. model. The enthalpy values for methyldopa in CO₂ were estimated at 34.12 kJ/mol using the Chrastil model and at 57.19 kJ/mol using the Bartle. Consequently, the solvation heat (ΔH_sol_) was found to be − 23.07 kJ/mol, representing the difference between the vaporization and total heats. A similar results has been reported in the solubility of mesalazine^53^, mebeverine^54^, alprazolam^55^ and erlotinib^56^.

**Fig. 3:**
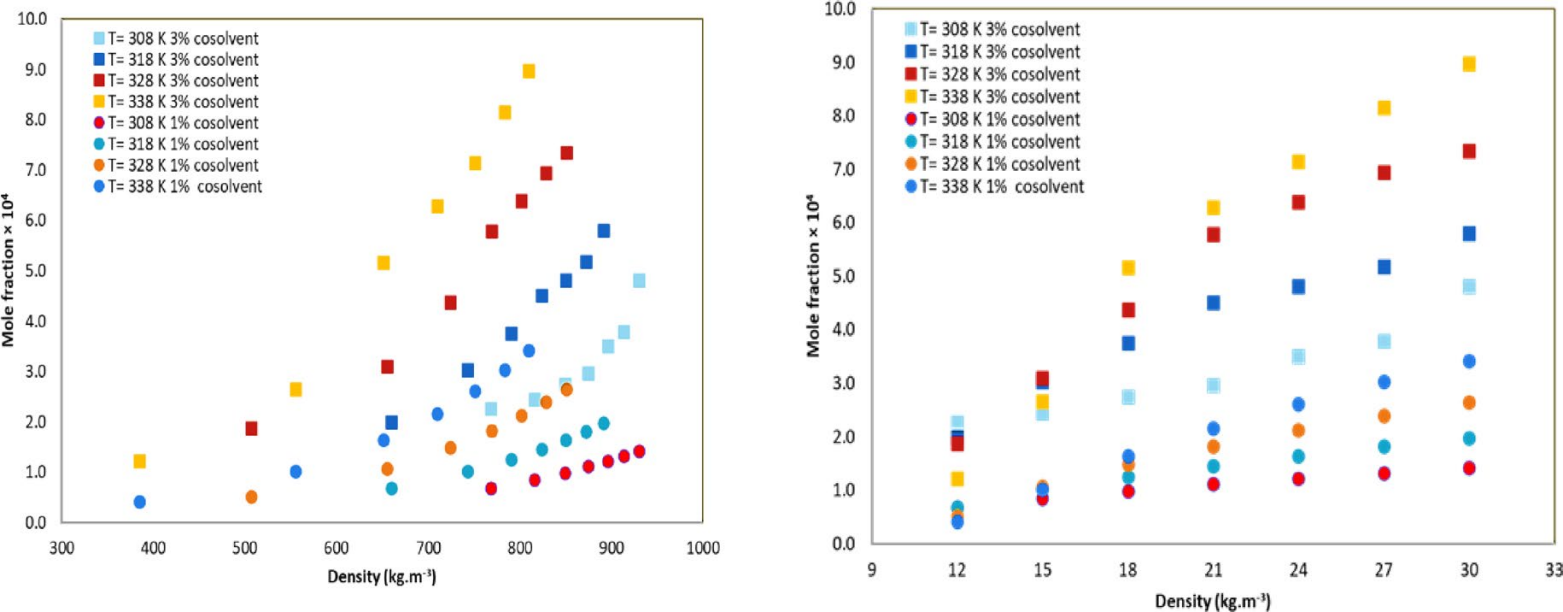
The methyldopa mole fraction in CO_2_ + ethanol vs. pressure and density.

As illustrated in Table 3; Fig. 3, the findings show how the solubility of methyldopa changes with density and pressure at different temperatures for the two ethanol concentrations. The study found that ethanol significantly improves methyldopa solubility in scCO₂, making ethanol a suitable choice for pharmaceutical applications due to its safety. Additionally, the enhancement factor (e) was calculated using the provided formula to quantify the impact of ethanol on solubility. This enhancement has been attributed to various factors, including polarity, temperature, and pressure effects.

17 $$\:e=\frac{{y}_{2}^{{\prime\:}}}{{y}_{2}}\times\:100=\frac{mole\:fraction\:of\:ternary}{mole\:fraction\:of\:binary}\times\:100$$

The highest and lowest effects of the co-solvent on solubility with 3 mol percent were observed 15.70 at 338 K and 12 MPa and 8.30 at 308 K and 30 MPa, respectively. The effect of co-solvents in increasing API solubility in scCO_2_ has been investigated in recent studies.

In a study spearheaded by Ahmar Khan et al.^57^, the solubility of baclofen, a muscle relaxant, was examined in pure scCO2 and in the presence of cosolvents, including ethanol and DMSO. The initial findings indicated low solubility for baclofen in pure scCO_2_, with values ranging from 1.62 × 10^−5^ to 2.30 × 10^−5^ mole fractions. The introduction of ethanol resulted in a substantial enhancement in solubility, ranging from 5.76 × 10^−5^ to 12.79 × 10^−5^ mole fractions. A similar enhancement was observed upon the introduction of DMSO, which increased the solubility range to 3.50 × 10 − 5 to 7.02 × 10–5 mol fractions. Machine learning approaches demonstrated over 99% accuracy in predicting solubility, underscoring their potential usefulness in future drug solubility studies.

Kloc and colleagues^58^ examined the solubility of naproxen and indomethacin in scCO2 and scCO2 with ethyl acetate. A comprehensive experimental and theoretical analysis was conducted by the research team, encompassing the assessment of solubility in a high-pressure view cell at temperatures spanning from 60 °C to 429 bar and pressures ranging from 150 to 429 bar. The addition of ethyl acetate to the solution of indomethacin led to a substantial enhancement in solubility, particularly at lower pressures, with an increase of two orders of magnitude being observed. The study employed PC-SAFT to model solubility with a high degree of accuracy.

Obaidullah’s^59^ study focused on Chlorothiazide, a diuretic characterized by its limited solubility and bioavailability. The research assessed its solubility in pure scCO2 and in ternary mixtures involving cosolvents (ethanol, DMSO, and acetone). The findings revealed that the supercritical solubilities of Chlorothiazide varied in the presence of these cosolvents: ethanol achieved solubility ranges from 1.115 × 10^−5^ to 11.895 × 10^−5^, DMSO ranged from 0.778 × 10^−5^ to 9.25 × 10^−5^, and acetone ranged from 0.668 × 10 − ^5^ to 9.04 × 10^−5^. The findings indicated that cosolvents notably augmented Chlorothiazide’s solubility, with ethanol exhibiting a particularly pronounced effect, increasing solubility by a range of 2.02 to 11.75 times. Alsawad et al.^60^ research focused on the solubility of febuxostat, a drug used to treat gout by reducing uric acid levels in the blood, in scCO_2_. Initial results show that the drug can dissolve in scCO2 in the absence of cosolvents, with a range of 0.05 × 10^−4^ to 7.42 × 10^−4^. The study then investigated the effect of three additional substances, namely ethanol, acetone and DMSO, on the solubility of the drug. The results showed that the presence of ethanol significantly increased the solubility of the drug. The solubility enhancement ranged from 2.4 to 3.8 times. The solubility range was from 0.180 × 10^−4^ to 26.658 × 10^−4^. Acetone and DMSO also increased solubility, but not to the same extent (by about 2 to 2.5 times) and with lower numbers (ranging from 0.120 × 10^−4^ to 14.810 × 10^−4^ and 0.108 × 10^−4^ to 14.366 × 10^−4^). The results also show that temperature has been demonstrated to substantially influence solubility. Studies indicate that increasing the temperature while keeping it constant results in enhanced methyldopa solubility in both systems.

**Table 3:** Methyldopa solubility in scCO_2_ + 1 and 3 mol % ethanol.

Temperature (K)	Pressure (MPa)	1 mol % ethanol			3 mol % ethanol		
		Mole fraction (y’_2_) × 10^4^	Standard Deviation ×10^4^	enhancement factor (e)	Mole fraction (y’_2_) × 10^4^	Standard Deviation ×10^4^	enhancement factor (e)
308	12	0.766	0.026	3.22	2.259	0.007	9.49
	15	0.768	0.027	2.65	2.456	0.009	8.47
	18	0.950	0.032	2.47	2.759	0.01	7.17
	21	1.038	0.035	2.39	2.964	0.010	6.83
	24	1.216	0.041	2.30	3.405	0.013	6.44
	27	1.377	0.047	2.21	3.793	0.011	6.08
	30	1.423	0.048	1.79	4.677	0.012	5.88
318	12	0.701	0.024	3.91	2.001	0.007	11.18
	15	1.065	0.036	3.30	3.044	0.008	9.42
	18	1.318	0.045	3.00	3.766	0.010	8.58
	21	1.578	0.053	2.74	4.510	0.011	7.82
	24	1.687	0.057	2.42	4.819	0.012	6.91
	27	1.815	0.061	2.39	5.187	0.016	6.83
	30	2.031	0.069	2.35	5.803	0.019	6.72
328	12	0.622	0.021	4.21	1.872	0.005	12.65
	15	0.914	0.031	3.35	3.097	0.008	11.35
	18	1.536	0.052	3.17	4.387	0.011	9.06
	21	2.026	0.069	2.95	5.789	0.014	8.41
	24	2.237	0.076	2.88	6.392	0.015	8.22
	27	2.434	0.082	2.72	6.953	0.017	7.76
	30	2.558	0.087	2.62	7.361	0.019	7.55
338	12	0.405	0.013	5.19	1.225	0.003	15.70
	15	0.925	0.031	4.69	2.660	0.009	13.50
	18	1.808	0.061	3.46	5.165	0.014	9.88
	21	2.204	0.075	3.04	6.298	0.016	8.70
	24	2.502	0.085	2.96	7.149	0.019	8.46
	27	2.858	0.097	2.91	8.166	0.021	8.32
	30	3.143	0.107	2.90	8.979	0.023	8.30

The phenomenon of a retrograde region, where solubility diminishes with rising temperature under constant pressure, was observed. The study identified crossover pressures, which are indicative of points where solubility behavior changes. These pressures were noted at approximately 17 MPa for binary systems and 14 MPa for ternary systems. The crossover point is the pressure at which solubility isotherms converge. This pressure typically ranges from 10 to 20 MPa. This marks a shift in the effects of temperature. Below this point, higher temperatures reduce solubility due to decreased scCO₂ density. Above this point, solubility increases due to enhanced solute vapor pressure. This phenomenon is driven by a thermodynamic balance in which the temperature derivative of solubility is zero. Co-solvents, such as ethanol, lower the crossover pressure by increasing polarity. For example, Riluzole^61^ shows a crossover at 18–20 MPa, alprazolam^55^ at 18–21 MPa, fexofenadine^62^at 16–18 MPa, and ibrutinib^24^ at 15–17 MPa. Retrograde vaporization occurs below the crossover point, where solubility decreases with temperature. The crossover region is influenced by solute properties, such as the enthalpy of sublimation and the partial molar enthalpy in the supercritical phase. The crossover point reflects the balance between density-driven (enthalpic) and vapor pressure-driven (entropic) effects. As discussed in several articles, co-solvents modify solvation energy and shift the equilibrium^42,55,57,60,63,64^.

Due to the low polarity of CO₂, it is generally ineffective at dissolving polar compounds. However, the introduction of co-solvents like ethanol (polar) can significantly enhance polarity of scCO₂. The chemical structure of methyldopa, which includes OH, NH_2_, and C = O functional groups, contributes to its polarity, thereby increasing its solubility in ternary systems, particularly in high-density solvents. Additionally, adding ethanol increased the density of CO_2_, which increased its solubility potential.

Ethanol is frequently chosen as a co-solvent in supercritical CO₂ systems due to its lower toxicity, regulatory acceptance, and environmental sustainability compared to methanol and acetone. Its favorable safety profile allows for its use in a broader range of applications in the pharmaceutical, food, and biomedical industries, aligning with international guidelines^65^. Unlike methanol, which is produced from fossil fuels and raises environmental concerns, ethanol is an eco-friendly option because it is biodegradable and derived from renewable sources. Ethanol enhances the solubility of polar compounds and is compatible with pharmaceutical processes such as particle formation. These properties further underscore its suitability. Additionally, ethanol is economical because it is readily available and cost-effective, making it ideal for large-scale use. In contrast, methanol’s toxicity limits its regulatory approval, and acetone’s lower polarity and less favorable process compatibility reduce its utility^66^. Overall, ethanol’s balanced physicochemical properties, favorable safety profile, environmental benefits, and regulatory approval make it the preferred cosolvent in supercritical CO₂ applications.

Furthermore, the study also examined the efficacy of various models that utilize density to predict solubility. These models were utilized to establish links between various solutes. Two criteria were employed to assess the models’ performance. The AARD and R^2^ metrics were used to evaluate the performance of the models.

18 $$\:AARD\%=\frac{1}{\:N\:}{\sum\:}_{i=1}^{n}\left(\left|\frac{{y}_{i,cal}-{y}_{i,exp}}{{y}_{i,exp}\:}\right|\right)\times\:100\:\%$$

19 $$R^2=1-\frac{\sum_{n=1}^{N}(y_n^{exp}-{y_n^{calc})^2}}{\sum_{n=1}^{N}(y_n^{exp}-{\overline{y})^2}}$$

**Fig. 4:**
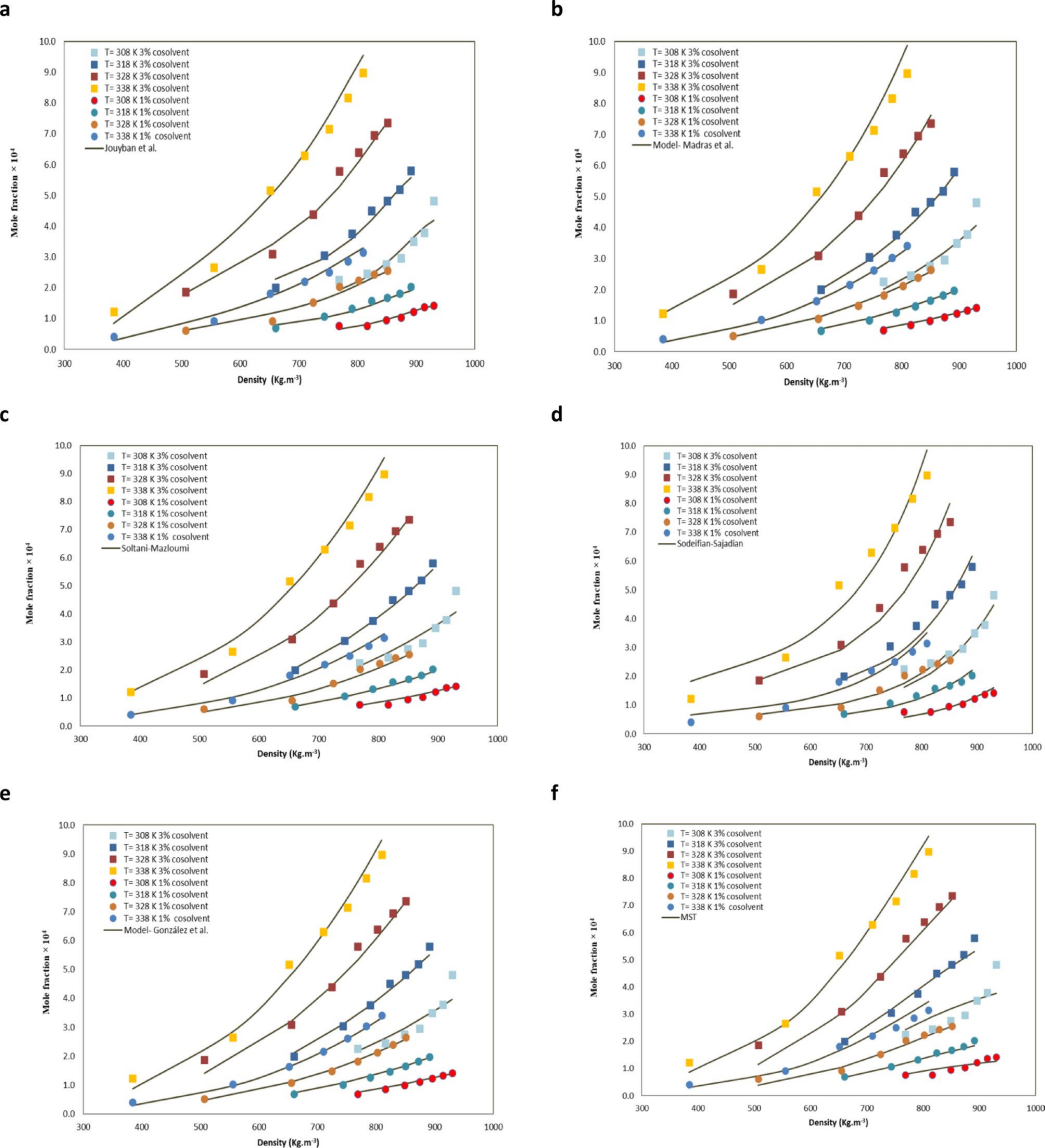
Modeling of methyldopa solubility in CO_2_ + ethanol: (**a**) Jouyban et al., (**b**) Madras et al., (**c**) Soltani-Mazlumi (**d**) Sodeifian-Sajadian (**e**) Gonzales et al., and (**f**) MST.

The adjustable parameters of the models were estimated using a simulated annealing algorithm. MATLAB version 2021b was used for the software. The AARD of Soltani-Mazloumi, Jouyban et al., MST, Sodeifian-Sajadian, Madras and González et al. models were achieved at 8.70, 10.18, 5.94, 7.87, 6.11, and 6.74, respectively (Table 3). The Soltani-Mazloumi model, which incorporates four adjustable parameters, demonstrated efficacy in the analysis of solubility data. The findings substantiated that all semi-empirical correlations effectively predicted methyldopa solubility in supercritical fluids. The experimental and calculated solubility data for ternary systems are presented in Table 3; Fig. 4.

**Table 4:** Results of the models of Methyldopa in ethanol + scCO_2_.

Model	a_0_	a_1_	a_2_	a_3_	a_4_	a_5_	a_6_	AARD	*R* ^2^	AICc
Gonzales	3.27	0.955	−4547.2	−12.075				6.74	0.993	−554.4
Sodeifian -Sajadian	−1.77	−0.5815	0.0177	0.9367				10.18	0.986	−537.2
MST	−9402.5	2.68	18.16	17195.1				8.64	0.990	−543.2
Soltani Mazloumi	−1.306	−5668.5	1.215	0.1621	0.972			5.94	0.995	−562.8
Madras et al.	2.903	1.295	0.003	−4908.2	0.424	1.44	−0.477	6.11	0.994	−559.7
Jouyban	−63.57	53.19	−0.0144	−0.00195	0.000479	−0.0059	9.51	7.87	0.992	−547.4

Obaidullah’s^59^ also used empirical models and an artificial neural network (ANN) approach to predict these solubility results. The Jouyban model was found to be the most accurate in correlating solubility data across all cosolvents. The ANN model demonstrated a high degree of accuracy with a mean absolute relative deviation percentage of 3.207% and a coefficient of determination (R^2^) of 0.993 for predicting solubility in different solvent systems. Bitencourt’s^67^ research concentrated on the solubility of solid solutes in scCO2, with and without the presence of organic cosolvents. To this end, the Cubic Plus Association equation of state (CPA-EoS) was utilized. A comprehensive understanding of solubility is imperative for the optimization of extraction, fractionation, and purification processes in diverse industrial sectors, including food, pharmaceuticals, and materials. The research evaluated the solubility of 12 different solid solutes across 19 distinct systems, examining pressures between 8 and 40 MPa, temperatures from 308 K to 353 K, and cosolvent concentrations ranging from 0.73 to 10 mol%. The findings yielded an average logarithmic deviation of 0.47 between the experimental data and predictions made using CPA-EoS, signifying enhanced accuracy compared to the previously employed PR + COSMOSAC method. Additionally, the CPA-EoS exhibited efficacy in estimating the impacts of temperature, the type and concentration of cosolvents and pressure on solubility. This research underscores the potential of CPA-EoS in enhancing the understanding and design of processes involving solid solutes in supercritical environments (Table 4).

Figures S4 and Tables 3S show the correlation analysis of the EoS using two interaction parameters (vdW2) at temperatures of 308, 318, 328, and 338 K. Using the PR equation with the vdW2 mixing rule produced better results: an AARD% of 10.71, R² of 0.975 and AICc of −444.5. The results clearly show that PR model confirms the data well. The model’s tunable parameters were calculated using a simulated annealing method.

## Conclusion

This study aims to evaluate the solubility of methyldopa in two experimental supercritical fluid systems: pure scCO₂ and scCO₂ with 1–3 mol percent ethanol as a co-solvent. The study was conducted at temperatures ranging from 308 to 338 K and pressures ranging from 12 to 30 MPa. In the binary system, the mole fraction of methyldopa ranged from 0.078 × 10^−4^ to 1.082 × 10^−4^, indicating the limitations of scCO₂ in dissolving this active API. In contrast, the ternary system with ethanol as a cosolvent exhibited substantially greater solubility, with mole fractions ranging from 1.880 × 10^−5^ to 1.237 × 10^−4^ at 1 and 3 mol percent, respectively. This increase in solubility is attributed to favorable intermolecular interactions, such as dipole-induced dipole and dipole-dipole interactions, between the methyldopa and ethanol molecules in the ternary system. Findings showed that maximum solubility was achieved at 12 MPa pressure and 338 K temperature with 1 and 3 mol% ethanol, resulting in 5.19 and 15.70 fold increases, respectively. These results show that integrating ethanol into the supercritical fluid system substantially enhances methyldopa’s solubility. The maximum mole fraction of methyldopa, 8.979 × 10^−4^, was achieved with ethanol under these conditions. These results highlight the critical role of co-solvents in improving methyldopa solubility in supercritical fluid environments.

## Supplementary Information

Below is the link to the electronic supplementary material.

Supplementary Material 1

## Data Availability

All data generated or analysed during this study are included in this published article.

## References

[1] Wiciński, M., Malinowski, B., Puk, O., Socha, M. & Słupski, M. Methyldopa as an inductor of postpartum depression and maternal blues: A review. *Biomed. Pharmacother.* **127**, 110196. 10.1016/j.biopha.2020.110196 (2020). 32413670 10.1016/j.biopha.2020.110196

[2] Myhre, E., Rugstad, H. E. & Hansen, T. Clinical pharmacokinetics of Methyldopa. *Clin. Pharmacokinet.* **7**, 221–233. 10.2165/00003088-198207030-00003 (1982). 7047042 10.2165/00003088-198207030-00003

[3] Junyaprasert, V. B. & Morakul, B. Nanocrystals for enhancement of oral bioavailability of poorly water-soluble drugs. *Asian J. Pharm. Sci.* **10**, 13–23. 10.1016/j.ajps.2014.08.005 (2015).

[4] Khadka, P. et al. Pharmaceutical particle technologies: an approach to improve drug solubility, dissolution and bioavailability. *Asian J. Pharm. Sci.* **9**, 304–316 (2014).

[5] Krishnaiah, Y. S. Pharmaceutical technologies for enhancing oral bioavailability of poorly soluble drugs. *J. Bioequiv Availab.* **2**, 28–36 (2010).

[6] Esfandiari, N. & Sajadian, S. A. CO2 utilization as gas antisolvent for the pharmaceutical micro and nanoparticle production: A review. *Arabian J. Chemistry*, **104164** (2022).

[7] Kankala, R. K., Xu, P. Y., Chen, B. Q., Wang, S. B. & Chen, A. Z. Supercritical fluid (SCF)-assisted fabrication of carrier-free drugs: an eco-friendly welcome to active pharmaceutical ingredients (APIs). *Advanced Drug Delivery Reviews*, **113846** (2021). 10.1016/j.addr.2021.113846 34197896

[8] Padrela, L. et al. Supercritical carbon dioxide-based technologies for the production of drug nanoparticles/nanocrystals–A comprehensive review. *Advanced Drug Delivery Reviews* (2018). 10.1016/j.addr.2018.07.010 30026127

[9] Knez, Ž. et al. Industrial applications of supercritical fluids: A review. *Energy* **77**, 235–243 (2014).

[10] Sodeifian, G., Sajadian, S. A., Ardestani, N. S. & Razmimanesh, F. Production of Loratadine drug nanoparticles using ultrasonic-assisted rapid expansion of supercritical solution into aqueous solution (US-RESSAS). *J. Supercrit. Fluids*. 10.1016/j.supflu.2018.11.007 (2018).

[11] Fattahi, A. et al. Preparation and characterization of Simvastatin nanoparticles using rapid expansion of supercritical solution (RESS) with trifluoromethane. *J. Supercrit. Fluids*. **107**, 469–478 (2016).

[12] Türk, M. & Bolten, D. Polymorphic properties of micronized mefenamic acid, nabumetone, Paracetamol and tolbutamide produced by rapid expansion of supercritical solutions (RESS). *J. Supercrit. Fluids*. **116**, 239–250 (2016).

[13] Sodeifian, G., Alwi, R. S., Razmimanesh, F. & Abadian, M. Solubility of dasatinib monohydrate (anticancer drug) in supercritical CO2: experimental and thermodynamic modeling. *J. Mol. Liq.* **346**, 117899 (2022).

[14] Pishnamazi, M. et al. Chloroquine (antimalaria medication with anti SARS-CoV activity) solubility in supercritical carbon dioxide. *J. Mol. Liq.* **322**, 114539 (2021). 33071399 10.1016/j.molliq.2020.114539 PMC7550982

[15] Wichianphong, N. & Charoenchaitrakool, M. Statistical optimization for production of mefenamic acid–nicotinamide cocrystals using gas anti-solvent (GAS) process. *J. Ind. Eng. Chem.* **62**, 375–382 (2018).

[16] Arabgol, F., Amani, M., Ardestani, N. S. & Sajadian, S. A. Experimental and thermodynamic investigation of Gemifloxacin solubility in supercritical CO2 for the production of nanoparticles. *J. Supercrit. Fluids*. **206**, 106165 (2024).

[17] Ansari, E., Honarvar, B., Sajadian, S. A., Aboosadi, Z. A. & Azizi, M. Solubility of Aripiprazole in supercritical carbon dioxide: Experimental and modeling evaluations. (2023). 10.1038/s41598-023-40537-3 PMC10435544 37591914

[18] Alwi, R. S. et al. Experimental study and thermodynamic modeling of clonazepam solubility in supercritical carbon dioxide. *Fluid. Phase. Equilibria*. **574**, 113880. 10.1016/j.fluid.2023.113880 (2023).

[19] Honarvar, B., Sajadian, S. A., Rojas, A., Galotto, M. J. & Jouyban, A. Solubility and thermodynamic modeling of sildenafil citrate in supercritical carbon dioxide. *Fluid. Phase. Equilibria*. **566**, 113677. 10.1016/j.fluid.2022.113677 (2023).

[20] Esfandiari, N. & Sajadian, S. A. Solubility of lacosamide in supercritical carbon dioxide: an experimental analysis and thermodynamic modeling. *J. Mol. Liq.* **360**, 119467 (2022). 10.1016/j.supflu.2022.105539 PMC8815272 35136283

[21] Sodeifian, G., Sajadian, S. A. & Derakhsheshpour, R. Experimental measurement and thermodynamic modeling of Lansoprazole solubility in supercritical carbon dioxide: application of SAFT-VR EoS. *Fluid. Phase. Equilibria*. **507**, 112422 (2020).

[22] Sodeifian, G., Saadati Ardestani, N., Sajadian, S. A., Golmohammadi, M. R. & Fazlali, A. Prediction of solubility of sodium valproate in supercritical carbon dioxide: experimental study and thermodynamic modeling. *J. Chem. Eng. Data*. **65**, 1747–1760 (2020).

[23] Sodeifian, G., Razmimanesh, F. & Sajadian, S. A. Solubility measurement of a chemotherapeutic agent (Imatinib mesylate) in supercritical carbon dioxide: assessment of new empirical model. *The J. Supercritical Fluids* (2019).

[24] Sodeifian, G., Nasri, L., Razmimanesh, F. & Nooshabadi, M. A. Solubility of ibrutinib in supercritical carbon dioxide (Sc-CO2): data correlation and thermodynamic analysis. *J. Chem. Thermodyn.* **182**, 107050 (2023).

[25] Sodeifian, G., Garlapati, C., Razmimanesh, F. & Nateghi, H. Solubility measurement and thermodynamic modeling of Pantoprazole sodium sesquihydrate in supercritical carbon dioxide. *Sci. Rep.* **12**, 7758 (2022). 35546179 10.1038/s41598-022-11887-1 PMC9095875

[26] Pishnamazi, M. et al. Measuring solubility of a chemotherapy-anti cancer drug (busulfan) in supercritical carbon dioxide. *J. Mol. Liq.* **317**, 113954 (2020).

[27] Bian, X. Q., Zhang, Q., Du, Z. M., Chen, J. & Jaubert, J. N. A five-parameter empirical model for correlating the solubility of solid compounds in supercritical carbon dioxide. *Fluid. Phase. Equilibria*. **411**, 74–80 (2016).

[28] Bartle, K. D., Clifford, A., Jafar, S. & Shilstone, G. Solubilities of solids and liquids of low volatility in supercritical carbon dioxide. *J. Phys. Chem. Ref. Data*. **20**, 713–756 (1991).

[29] Méndez-Santiago, J. & Teja, A. S. The solubility of solids in supercritical fluids. *Fluid. Phase. Equilibria*. **158**, 501–510 (1999).

[30] Mendez-Santiago, J. & Teja, A. S. Solubility of solids in supercritical fluids: consistency of data and a new model for cosolvent systems. *Ind. Eng. Chem. Res.* **39**, 4767–4771 (2000).

[31] Del Valle, J. M. & Aguilera, J. M. An improved equation for predicting the solubility of vegetable oils in supercritical carbon dioxide. *Ind. Eng. Chem. Res.* **27**, 1551–1553 (1988).

[32] Garlapati, C. & Madras, G. New empirical expressions to correlate solubilities of solids in supercritical carbon dioxide. *Thermochim Acta*. **500**, 123–127 (2010).

[33] Kumar, S. K. & Johnston, K. P. Modelling the solubility of solids in supercritical fluids with density as the independent variable. *J. Supercrit. Fluids*. **1**, 15–22 (1988).

[34] González, J. C., Vieytes, M. R., Botana, A. M., Vieites, J. M. & Botana, L. M. Modified mass action law-based model to correlate the solubility of solids and liquids in entrained supercritical carbon dioxide. *J. Chromatogr. A*. **910**, 119–125 (2001). 11263564 10.1016/s0021-9673(00)01120-1

[35] Sodeifian, G., Sajadian, S. A. & Ardestani, N. S. Optimization of essential oil extraction from launaea acanthodes boiss: utilization of supercritical carbon dioxide and cosolvent. *J. Supercrit. Fluids*. **116**, 46–56 (2016).

[36] Tabernero, A., de Melo, S. V., Mammucari, R., del Valle, E. M. & Foster, N. Modelling solubility of solid active principle ingredients in sc-CO 2 with and without cosolvents: A comparative assessment of semiempirical models based on chrastil’s equation and its modifications. *J. Supercrit. Fluids*. **93**, 91–102 (2014).

[37] Tang, C., Guan, Y. X., Yao, S. J. & Zhu, Z. Q. Solubility of dexamethasone in supercritical carbon dioxide with and without a cosolvent. *J. Chem. Eng. Data*. **59**, 3359–3364 (2014).

[38] Hosseini, M. H., Alizadeh, N. & Khanchi, A. R. Effect of menthol as solid cosolvent on the solubility enhancement of clozapine and lamorigine in supercritical CO2. *J. Supercrit. Fluids*. **55**, 14–22 (2010).

[39] Tsai, C. C., Lin, H. & Lee, M. J. Solubility of CI disperse Violet 1 in supercritical carbon dioxide with or without cosolvent. *J. Chem. Eng. Data*. **53**, 2163–2169 (2008).

[40] Huang, Z., Kawi, S. & Chiew, Y. Solubility of cholesterol and its esters in supercritical carbon dioxide with and without cosolvents. *J. Supercrit. Fluids*. **30**, 25–39 (2004).

[41] Rojas, A. et al. Solubility of Oxazepam in supercritical carbon dioxide: experimental and modeling. *Fluid. Phase. Equilibria*. **585**, 114165. 10.1016/j.fluid.2024.114165 (2024).

[42] Askarizadeh, M., Esfandiari, N., Honarvar, B., Sajadian, S. A. & Azdarpour, A. Solubility of Teriflunomide in supercritical carbon dioxide and co-solvent investigation. *Fluid. Phase. Equilibria*. **590**, 114284. 10.1016/j.fluid.2024.114284 (2025).

[43] Sodeifian, G., Sajadian, S. A., Razmimanesh, F. & Hazaveie, S. M. Solubility of ketoconazole (antifungal drug) in SC-CO2 for binary and ternary systems: measurements and empirical correlations. *Sci. Rep.* **11**, 1–13 (2021). 33824375 10.1038/s41598-021-87243-6 PMC8024397

[44] Soltani, S. & Mazloumi, S. H. A new empirical model to correlate solute solubility in supercritical carbon dioxide in presence of co-solvent. *Chem. Eng. Res. Des.* **125**10.1016/j.cherd.2017.07.006 (2017).

[45] Garlapati, C. & Madras, G. Solubilities of solids in supercritical fluids using dimensionally consistent modified solvate complex models. *Fluid Phase Equilib.* **283**10.1016/j.fluid.2009.05.013 (2009).

[46] Jouyban, A., Khoubnasabjafari, M. & Chan, H. K. Modeling the entrainer effects on solubility of solutes in supercritical carbon dioxide. *Chem. Pharm. Bull.* **53**10.1248/cpb.53.290 (2005). 10.1248/cpb.53.290 15744100

[47] Chrastil, J. Solubility of solids and liquids in supercritical gases. *J. Phys. Chem.* **86**, 3016–3021 (1982).

[48] Soltani, S. & Mazloumi, S. H. A new empirical model to correlate solute solubility in supercritical carbon dioxide in presence of co-solvent. *Chem. Eng. Res. Des.* **125**, 79–87 (2017).

[49] Hozhabr, S. B., Mazloumi, S. H. & Sargolzaei, J. Correlation of solute solubility in supercritical carbon dioxide using a new empirical equation. *Chem. Eng. Res. Des.* **92**, 2734–2739 (2014).

[50] Jouyban, A., Khoubnasabjafari, M. & Chan, H. K. Modeling the entrainer effects on solubility of solutes in supercritical carbon dioxide. *Chem. Pharm. Bull.* **53**, 290–295 (2005). 10.1248/cpb.53.290 15744100

[51] Immirzi, A. & Perini, B. Prediction of density in organic crystals. *Acta Crystallogr. Sect. A*. **33**10.1107/S0567739477000448 (1977).

[52] Wali, A. F. et al. Determination of the solubility of Methyldopa in supercritical carbon dioxide for drug delivery applications: thermal analysis. *Sci. Rep.* **15**, 923 (2025). 39762304 10.1038/s41598-024-84263-w PMC11704008

[53] Sajadian, S. A. et al. Mesalazine solubility in supercritical carbon dioxide with and without cosolvent and modeling. *Sci. Rep.* **15**, 3870. 10.1038/s41598-025-86004-z (2025). 39890916 10.1038/s41598-025-86004-z PMC11785961

[54] Sajadian, S. A., Esfandiari, N., Ardestani, S., Amani, N., Estévez, L. A. & M. & Measurement and modeling of the solubility of Mebeverine hydrochloride in supercritical carbon dioxide. *Chem. Eng. Technol.* **47**, 811–821 (2024).

[55] Sajadian, S. A. et al. Solubility Measurement and Correlation of Alprazolam in Carbon Dioxide with/without Ethanol at Temperatures from 308 to 338 K and Pressures from 120 to 300 bar. *J. Chem. Eng. Data*. **69**, 1718–1730. 10.1021/acs.jced.3c00587 (2024).

[56] Bazaei, M., Honarvar, B., Esfandiari, N., Sajadian, S. A. & Arab Aboosadi, Z. Preparation of erlotinib hydrochloride nanoparticles (anti-cancer drug) by RESS-C method and investigating the effective parameters. *Sci. Rep.* **14**10.1038/s41598-024-64477-8 (2024). 10.1038/s41598-024-64477-8 PMC11213895 38942802

[57] Khan, M. A. et al. Study of Baclofen solubility in supercritical CO2 with and without cosolvents: experimental analysis, thermodynamic evaluation, and machine learning methods. *J. Chem. Eng. Data*. **70**, 953–971. 10.1021/acs.jced.4c00407 (2025).

[58] Kloc, A. P., Danzer, A. & Sadowski, G. Solubility of Naproxen and indomethacin in supercritical carbon dioxide/ethyl acetate mixtures. *J. Supercrit. Fluids*. **200**, 105990. 10.1016/j.supflu.2023.105990 (2023).

[59] Obaidullah, A. J. Thermodynamic and experimental analysis of drug nanoparticles Preparation using supercritical thermal processing: solubility of Chlorothiazide in different Co-solvents. *Case Stud. Therm. Eng.* **49**, 103212. 10.1016/j.csite.2023.103212 (2023).

[60] Alsawad, O. S. et al. Investigating the influence of cosolvents on the solubility of febuxostat in supercritical CO2: experimental analysis and artificial intelligence study. *J. Chem. Eng. Data*. **69**, 2569–2584. 10.1021/acs.jced.4c00208 (2024).

[61] Abadian, M., Sodeifian, G., Razmimanesh, F. & Zarei Mahmoudabadi, S. Experimental measurement and thermodynamic modeling of solubility of riluzole drug (neuroprotective agent) in supercritical carbon dioxide. *Fluid Phase Equilib* 567 (2023).

[62] Sodeifian, G., Bagheri, H., Arbab Nooshabadi, M., Razmimanesh, F. & Roshanghias, A. Experimental solubility of Fexofenadine hydrochloride (antihistamine) drug in SC-CO2: evaluation of cubic equations of state. *J Supercrit Fluids* **200** (2023).

[63] Askarizadeh, M., Esfandiari, N., Honarvar, B., Ali Sajadian, S. & Azdarpour, A. Binary and ternary approach of solubility of Rivaroxaban for Preparation of developed nano drug using supercritical fluid. *Arab. J. Chem.* **17**10.1016/j.arabjc.2024.105707 (2024).

[64] Li, B. et al. Cosolvent effect on the solubility of ammonium benzoate in supercritical carbon dioxide. *J. Chem. Eng. Data*. **67**, 689–694. 10.1021/acs.jced.1c00829 (2022).

[65] Connelly, J. ICH Q3C impurities: guideline for residual solvents. *ICH Qual. Guidelines: Implement. Guide*, 199–232 (2017).

[66] Valenzuela, L. M., Reveco-Chilla, A. G. & del Valle, J. M. Modeling solubility in supercritical carbon dioxide using quantitative structure–property relationships. *J. Supercrit. Fluids*. **94**, 113–122 (2014).

[67] Bitencourt, R. G., Palma, A. M., Coutinho, J. A., Cabral, F. A. & Meirelles, A. J. Prediction of solid solute solubility in supercritical CO2 with cosolvents using the CPA EoS. *Fluid. Phase. Equilibria*. **482**, 1–10 (2019).

