# Supplementary material for: Enhancing methyldopa solubility via green supercritical fluid techniques using ethanol co-solvent
Source: Sci Rep. 2025 Aug 13;15:29615. doi: 10.1038/s41598-025-15596-3 (PMC12344082; doi:10.1038/s41598-025-15596-3)
Supplement: Supplementary file 1 — Supplementary Material 1 [file 41598_2025_15596_MOESM1_ESM.docx]

**Enhancing Methyldopa Solubility via Green Supercritical Fluid Techniques Using Ethanol Co-Solvent**

Hadil Faris Alotaibi^1,*^, Suleiman Ibrahim Mohammad^2,3^, Asokan Vasudevan^4^, Suranjana V. Mayani^5^, Suhas Ballal^6^, Munthar Kadhim Abosaoda^7, 8^, Abhayveer Singh^9^, Subhashree Ray^10^, Atreyi Pramanik^11^

^1^Department of Pharmaceutical Sciences, College of Pharmacy, Princess Nourah Bint AbdulRahman University, Riyadh 11671, Saudi Arabia, Corresponding authors: [hadilfalotaibi@gmail.com](mailto:hadilfalotaibi@gmail.com)

^2^ Electronic Marketing and Social Media, Economic and Administrative Sciences, Zarqa University, Jordan.

^3^ Research follower, INTI International University, 71800 Negeri Sembilan, Malaysia.

^4^Faculty of Business and Communications, INTI International University, 71800 Negeri Sembilan, Malaysia.

^5^Marwadi University Research Center, Department of Chemistry, Faculty of Science, Marwadi University, Rajkot, Gujarat.

^6^Department of Chemistry and Biochemistry, School of Sciences, JAIN (Deemed to be University), Bangalore, Karnataka, India

^7^College of pharmacy, the Islamic University, Najaf, Iraq

^8^College of pharmacy, the Islamic University of Al Diwaniyah, Al Diwaniyah, Iraq

^9^Centre for Research Impact & Outcome, Chitkara University Institute of Engineering and Technology, Chitkara University, Rajpura, 140401, Punjab, India

^10^ Department of Biochemistry, IMS and SUM Hospital, Siksha 'O' Anusandhan (Deemed to be University), Bhubaneswar, Odisha-751003, India

^11^School of Applied and Life Sciences, Division of Research and Innovation, Uttaranchal University, Dehradun, Uttarakhand, India

Table S1. Preliminary tests.

| T | P | Time | Solubility (g/l) |
| --- | --- | --- | --- |
| 338 | 30 | 60 | 0.099±0.005 |
| 338 | 30 | 90 | 0.188±0.007 |
| 338 | 30 | 120 | 0.277±0.005 |
| 338 | 30 | 150 | 0.359±0.009 |
| 338 | 30 | 180 | 0.415±0.010 |
| 338 | 30 | 210 | 0.421±0.014 |
| 338 | 30 | 240 | 0.421±0.015 |

Table S2. Models for binary system (scCO_2_ + methyldopa)

| **Model name** | **Mathematical equation** |
| --- | --- |
| Chrastil | Ln S = a_0_ lnρ + a_1_ + a_2_/T |
| Kumar and Johnston | $\ln y_{2}=a_{0}+a_{1}\rho+\frac{a_{2}}{T}$ |
| Mendez-Santiago and Teja | $T ln\left( y_{2}P \right)=a_{0}+a_{1}\rho+a_{2}T$ |
| Bartle et al. | $\text{ln}\left( \frac{\text{y₂.p}}{\mathrm{Pref}} \right)\text{= }a_{0}\text{+}a_{1}\text{/T + a₂(ρ-}\text{ρ}\text{ref}\text{) }$ |

The results of the methyldopa-CO_2_ system correlated by semi-empirical models. Table S3.

| **Model** | $\mathbf{a}_{\mathbf{0}}$ | $\mathbf{a}_{\mathbf{1}}$ | $\mathbf{a}_{\mathbf{2}}$ | **AARD %** | **R_adj_** | **AIC** |
| --- | --- | --- | --- | --- | --- | --- |
| Chrastil | 4.78 | -4105.4 | -20.60 | 10.10 | 0.983 | -426.8 |
| Bartle et al. | 16.01 | 0.0086 | -6879.6 | 11.41 | 0.951 | -413.7 |
| MST | -8785.6 | 2.90 | 13.41 | 10.51 | 0.971 | -421.9 |
| K-J | -0.356 | 0.0059 | -4579.7 | 8.54 | 0.989 | -440.6 |

a_0_−a_2_, adjustable parameters of model.

Table S4. Correlation results of PR (scCO_2_ (1), methyldopa (2) and Ethanol (3))

| Parameter | T=308 K | T=318 K | T=328 K | T=338 K | overall |
| --- | --- | --- | --- | --- | --- |
| $\boldsymbol{k}_{\boldsymbol{12}}$ | -0.377 | -0.424 | -0.473 | -0.539 |  |
| $\boldsymbol{k}_{\boldsymbol{13}}$ | 0.086 | 0.086 | 0.086 | 0.086 |  |
| $\boldsymbol{k}_{\boldsymbol{23}}$ | -0.351 | -0.443 | -0.519 | -0.571 |  |
| $\boldsymbol{l}_{\boldsymbol{12}}$ | -0.529 | -0.619 | -0.773 | -0.857 |  |
| $\boldsymbol{l}_{\boldsymbol{13}}$ | 0.057 | 0.056 | 0.053 | 0.050 |  |
| $\boldsymbol{l}_{\boldsymbol{23}}$ | -0.467 | -0.469 | -0.471 | -0.476 |  |
| AARD% | 06.98 | 09.66 | 11.47 | 14.76 | 10.71 |
| R^2^ | 0.986 | 0.980 | 0.973 | 0.958 | 0.975 |
| AIC_C_ | -490.7 | -447.5 | -423.9 | -416.2 | -444.5 |


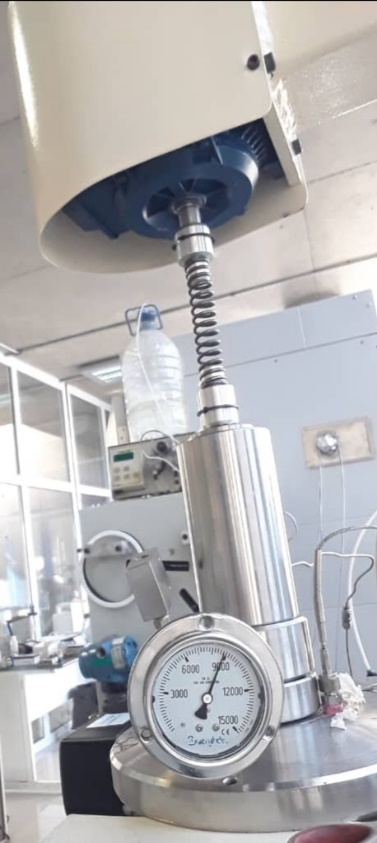


Figure S1. The cell and devices of the apparatus.


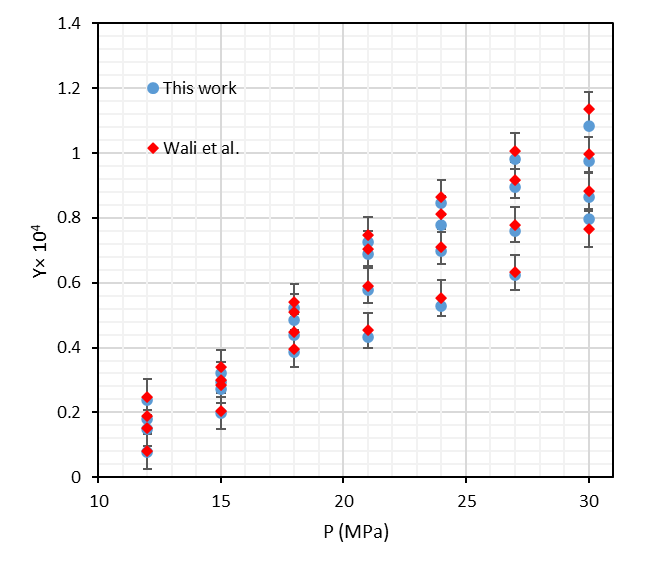


Figure S2. The comparison of data solubility of methyldopa in scCO_2_ in this work and Wali et al [53].

| 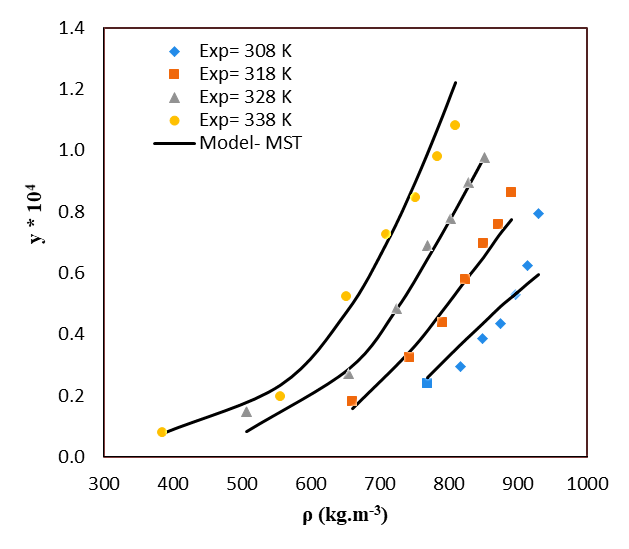 | 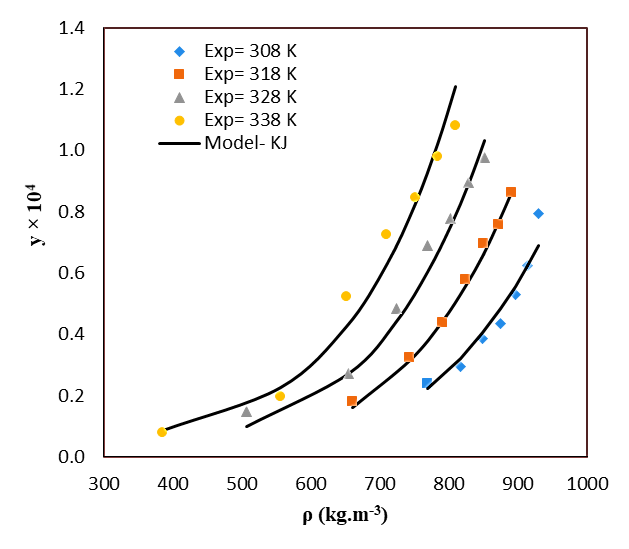 |
| --- | --- |
| 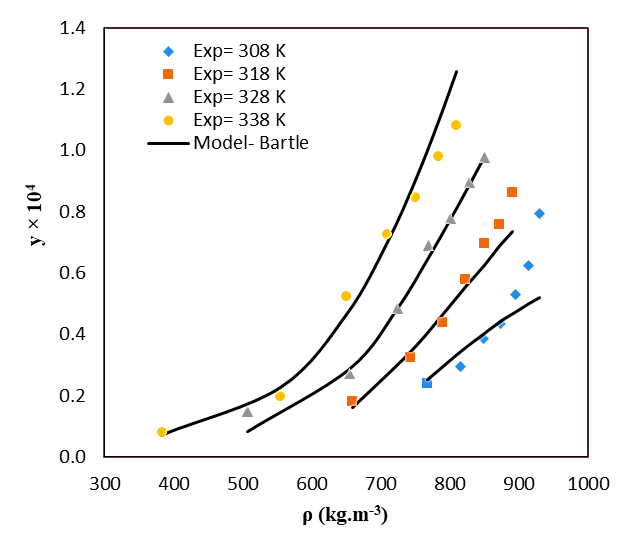 | 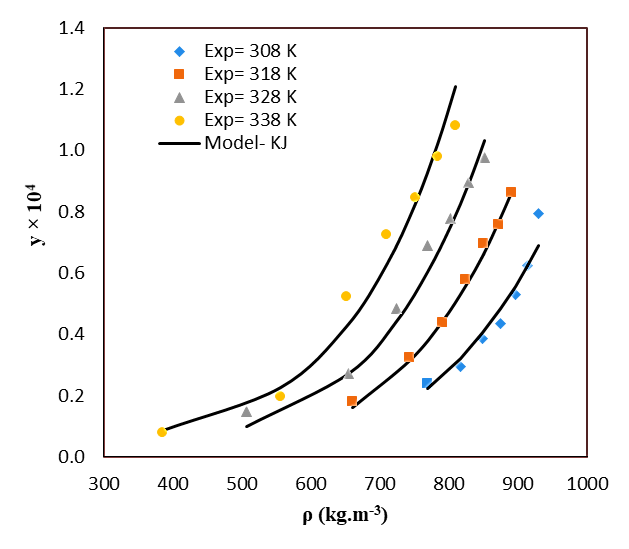 |

Figure S3. Comparison of experimental (points) and calculated (line) values solubility of methyldopa based on empirical models.


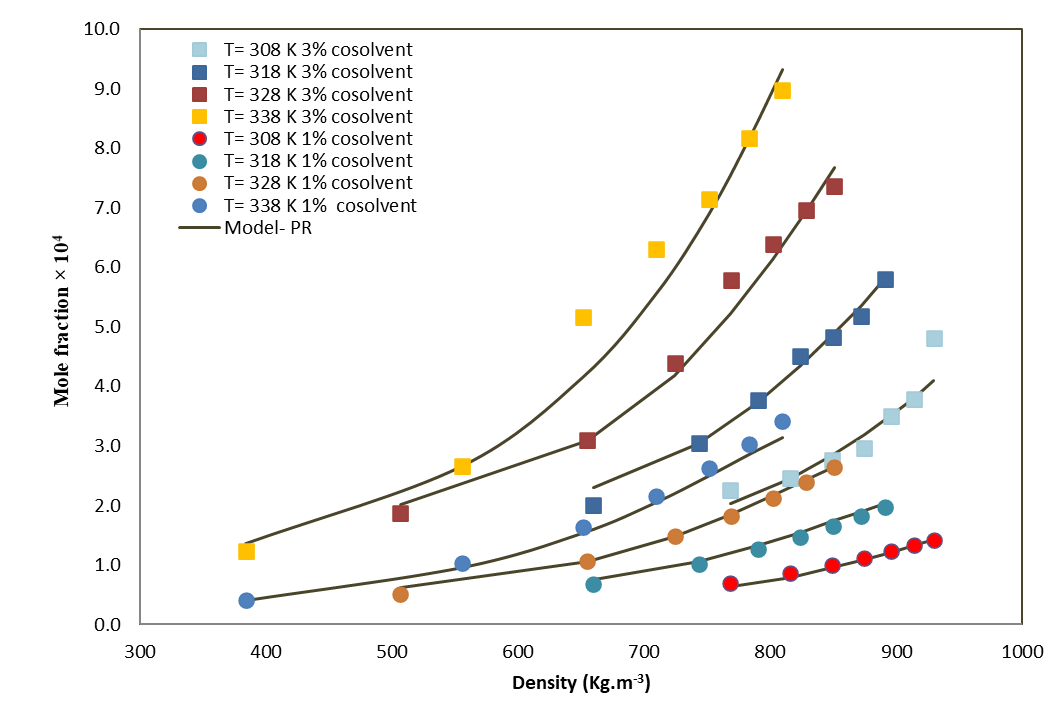


Figure S4. The results of PR ternary (CO_2_ + methyldopa + ethanol) systems.
